# Relationship between cardiovascular risk scores and subclinical coronary artery disease in people living with HIV

**DOI:** 10.1016/j.clinsp.2026.101013

**Published:** 2026-07-09

**Authors:** Carmen Ramos Alejos-Pita, Miriam Estébanez Muñoz, Tatiana Mata Forte, Juan José Reyes Luján, Matteo Romano, Edurne López Soberón, Francisco Javier Membrillo de Novales, Diego José Rodríguez Torres

**Affiliations:** a Department of Cardiology, Central Defense Hospital Gómez Ulla, Madrid, Spain; b University of Alcalá, Madrid, Spain; c CBRN and Department of Infectious Diseases, Central Defense Hospital Gómez Ulla, Madrid, Spain; d Research Section, Department of Education, Central Defense Hospital Gómez Ulla, Madrid, Spain; e Department of Radiology, Central Defense Hospital Gómez Ulla, Madrid, Spain

**Keywords:** HIV (human immunodeficiency virus), Cardiovascular disease, Subclinical atherosclerosis, Coronary computed tomography angiography, Cardiovascular risk stratification

## Abstract

**Background:**

People Living With HIV (PLWH) have an increased risk of cardiovascular disease, and conventional risk scores may underestimate subclinical coronary atherosclerosis. Coronary Computed Tomography Angiography (CCTA) enables direct plaque assessment and may improve cardiovascular risk stratification. This study evaluated factors associated with subclinical coronary disease and the performance of cardiovascular risk scores in high-risk PLWH.

**Methods:**

A prospective, single-center observational study was conducted, including 83 PLWH aged ≥ 40-years with sustained virological suppression between March 2022 and March 2023. Cardiovascular risk was estimated using Framingham, SCORE2, REGICOR, and D:A:D scores. Participants classified as high or very high risk underwent CCTA to detect subclinical coronary atherosclerosis. Clinical and laboratory variables associated with coronary disease were analyzed, and the predictive performance of risk scores was assessed using Receiver Operating Characteristic (ROC) curves.

**Results:**

The mean age was 59-years, and 92% of participants were male. Forty-seven individuals were classified as high cardiovascular risk. Subclinical coronary artery disease was detected in 52.2% of cases. Most lesions were mild and non-obstructive, although 48% of plaques showed vulnerability features. Fasting glucose levels were significantly associated with subclinical coronary disease (ρ = 0.47; p = 0.02). Among risk scores, the Framingham score demonstrated the highest predictive performance for CAC ≥ 100 (AUC = 0.78), followed by the reduced D:A:D score (AUC = 0.77)

**Conclusions:**

High-risk PLWH exhibit a high prevalence of subclinical coronary atherosclerosis. Fasting glucose was the main associated factor. Conventional cardiovascular risk scores showed moderate predictive ability, with the Framingham score performing best.

## Highlights

• Subclinical coronary atherosclerosis is highly prevalent in HIV (> 50%).
• CT angiography shows high burden of non-calcified and vulnerable plaque.
• Higher fasting glucose is linked to CAC and subclinical CAD despite control.
• Traditional cardiovascular risk scores show variable performance.
• Framingham Risk Score best identifies moderate-to-high coronary calcium burden.

## Introduction

The advent and widespread implementation of Antiretroviral Therapy (ART) have transformed Human Immunodeficiency Virus (HIV) infection into a chronic disease, leading to a marked improvement in survival. Consequently, as life expectancy among People Living with HIV (PLHIV) has increased, there has been a growing prevalence of non-AIDS-related comorbidities, with Cardiovascular Disease (CVD) emerging as one of the principal causes of morbidity and mortality in this population.^1^^,^^2^ Although considerable research has addressed this issue, the pathophysiological mechanisms responsible for the heightened cardiovascular risk in PLHIV are not yet fully elucidated. Available evidence suggests that PLHIV have a higher prevalence of subclinical atherosclerosis compared with the general population, even after accounting for traditional cardiovascular risk factors. In light of this increased cardiovascular burden, the identification of additional biomarkers that may improve cardiovascular risk stratification in PLHIV is of particular clinical relevance.^3^, ^4^, ^5^, ^6^, ^7^, ^8^, ^9^, ^10^

Cardiovascular risk prediction in clinical practice relies on several validated risk algorithms, including the Framingham Risk Score, SCORE2, and regionally adapted tools such as REGICOR, which was developed and calibrated in a Spanish population.^11^, ^12^, ^13^ Although these models are widely used in the general population, they were not specifically designed for PLHIV and do not incorporate HIV-related factors that may influence cardiovascular risk. .^8^, ^9^, ^10^, ^11^, ^12^, ^13^, ^14^ In contrast, the D:A:D risk equation was specifically developed and validated in PLHIV and includes HIV-specific variables, such as cumulative exposure to certain ART.^15^ However, despite its potential accuracy, the D:A:D score presents important limitations in routine clinical practice, as it requires detailed information on historical antiretroviral exposure, which may be unavailable or incomplete in patients with long-standing HIV infection or those who have received care in different healthcare systems.^14^, ^15^, ^16^ As a result, both general and HIV-specific risk scores may have practical or conceptual limitations when applied to real-world PLHIV populations, highlighting the need for complementary strategies to improve cardiovascular risk stratification.^17^^,^^18^

In this context, cardiovascular imaging has emerged as a valuable tool to refine risk stratification beyond traditional risk scores by providing a direct assessment of subclinical atherosclerosis. Coronary Computed Tomography Angiography (CCTA) allows for the non-invasive detection and characterization of coronary atherosclerotic plaque, including both calcified and non-calcified components, and has demonstrated strong prognostic value in asymptomatic individuals from the general population.^19^, ^20^, ^21^, ^22^ In subjects without known cardiovascular disease, CCTA has been shown to improve risk classification and identify individuals with a high atherosclerotic burden who may benefit from intensified preventive strategies.^23^, ^24^, ^25^, ^26^

CCTA-based studies in PLHIV have consistently shown an increased burden of non-calcified and mixed plaques, suggesting a distinct atherosclerotic phenotype that may not be fully captured by conventional risk scores. However, despite this growing body of evidence, the integration of coronary imaging into cardiovascular risk assessment in PLHIV remains limited in clinical practice, and data correlating risk estimates derived from commonly used cardiovascular risk scores with the presence of subclinical coronary artery disease detected by CCTA are still scarce.^6^^,^^27^, ^28^, ^29^, ^30^, ^31^, ^32^, ^33^ The present study aimed to identify the factors associated with the development of subclinical coronary artery disease and to evaluate which cardiovascular risk scores validated in clinical practice can predict its occurrence in PLHIV who have high or very high cardiovascular risk.

## Materials and methods

### Study design and population

**Fig. 1:**
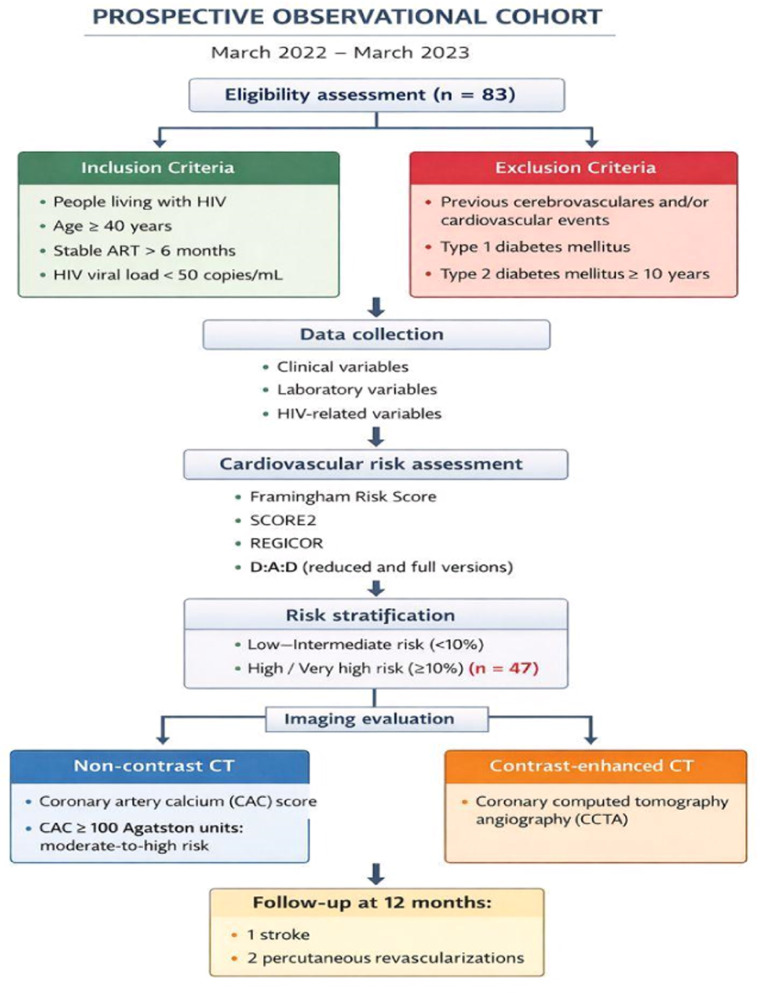
Study design and participant flow. Eligibility assessment, cardiovascular risk estimation, and imaging studies in people living with HIV. HIV, Human Immunodeficiency Virus; ART, Antiretroviral Therapy; CAC, Coronary Artery Calcium; CCTA, Coronary Computed Tomography Angiography.

This single-center, prospective, observational cohort study consecutively enrolled 83 PLHIV receiving routine clinical care at the monographic consultation of Hospital Gómez Ulla, Madrid, between March 2022 and March 2023. Inclusion criteria were: age ≥ 40-years, stable ART for >6-months, suppressed HIV viral load (< 50 copies/mL), and provision of written informed consent. Exclusion criteria included a history of cerebrovascular and/or cardiovascular events, type 1 diabetes mellitus, or type 2 diabetes mellitus of >10-years’ duration (or shorter duration in the presence of additional cardiovascular risk factors). Cardiovascular risk was assessed using different risk scores commonly applied in clinical practice: Framingham Risk Score, SCORE2, REGICOR, and D:A:D (Data Collection on Adverse Events of Anti-HIV Drugs). Those at high risk were offered CCTA, provided there were no contraindications to the test, such as severe renal insufficiency, contrast agent allergy, or refusal to provide informed consent (Fig. 1). The study protocol was approved by the local Ethics and Research Committee (protocol number 8_22), and the study was conducted and reported in accordance with the STROBE (Strengthening the Reporting of Observational Studies in Epidemiology) guidelines.

### Cardiovascular risk assessment

Cardiovascular risk was estimated using the following scores: Framingham Risk Score, SCORE2, REGICOR, and D:A:D (reduced and extended versions). Clinical and laboratory data were obtained from routine consultations; no additional measurements were performed for study purposes. Risk categories were defined according to published thresholds for each score:

• Framingham and REGICOR: low-intermediate (< 10%), high (10%–20%), very high (> 20%).
• SCORE2: < 50-years (< 2.5%, 2.5–7.5%, > 7.5%), 50–69 years (< 5%, 5–10%, > 10%), ≥ 70-years (< 7.5%, 7.5–15%, ≥ 15%).
• D:A:D: low-intermediate (< 10%), high (10–20%), very high (> 20%).

Not all patients had the necessary data to calculate the D:A:D scores, resulting in incomplete availability.

### Coronary computed tomography angiography (CCTA) and coronary artery calcium (CAC) scoring

Patients classified as high or very high risk by at least one score were eligible for CCTA.

• Non-contrast ECG (Electrocardiogram)-gated CT was performed to calculate CAC using a 64 × 1.5 mm detector CT scanner at 120 kV. The Agatston score was calculated to quantify baseline coronary atherosclerotic burden.
• Contrast-enhanced CCTA was subsequently performed using a 124-detector Philips scanner, with iodinated contrast (400 mg iodine/mL, 5 mL/s) followed by a 40 mL saline flush. Automatic bolus tracking was used, with acquisition triggered at 100 HU (Hounsfield Units) in the ascending aorta. Contrast volume ranged from 60 to 100 mL, adjusted for body weight and scan duration.

Images were post-processed using IntelliSpace Portal (Philips), generating curved multiplanar reformations and interactive oblique reconstructions. CAC and plaque characteristics were independently assessed by two blinded evaluators unaware of the cardiovascular risk scores, laboratory results, and clinical data. The following were considered plaque vulnerability features: positive remodeling, napkin-ring sign, spotty calcification, and low-attenuation plaque < 30 HU. Coronary stenosis severity was graded using CAD-RADS 0–5 according to the modified 17-segment model of the American Heart Association.

### Statistical analysis

A descriptive analysis was performed:

• Continuous variables, including age, BMI, glucose, lipids, triglycerides, apolipoprotein B, lipoprotein(a), CD4 count, CD4/CD8 ratio, estimated Glomerular Filtration Rate (eGFR, calculated using the Modification of Diet in Renal Disease [MDRD] equation), and risk scores, were expressed as mean ± standard deviation if normally distributed, or median (IQR) if non-normally distributed.
• Categorical variables (sex, race, family history of cardiovascular disease, type 2 diabetes, hypertension, smoking, statin use, hepatitis C serostatus, antiretroviral therapy regimen, and plaque characteristics) were reported as absolute counts and percentages.

Clinical and laboratory variables, as well as coronary plaque characteristics assessed by CT, were analyzed to evaluate their association with the Coronary Artery Calcium (CAC) score, treated as a continuous variable. Normality of continuous variables was assessed using the Shapiro-Wilk test. Associations with CAC, treated as a continuous variable, were evaluated using:

• Pearson correlation if both variables were normally distributed, or Spearman correlation (ρ) if not.
• Comparisons of CAC across categorical groups were performed using Mann-Whitney *U* or Kruskal-Wallis tests.

The predictive performance of cardiovascular risk scores for identifying subclinical atherosclerotic disease (defined as CAC ≥ 100 Agatston units) was assessed using Receiver Operating Characteristic (ROC) curve analysis, with calculation of Area Under the Curve (AUC) and 95% Confidence Intervals. A threshold of a coronary calcium score ≥ 100 Agatston units was established because patients with lower values are considered at very low or low risk of events (<5% over the next 5-years). The feasibility of score calculation was compared using Cochran’s *Q*-test. All analyses were two-sided, with statistical significance set at p < 0.05. Missing data were handled using complete-case analysis. All statistical analyses were conducted using R version 4.4.1 (R-Core Team, 2024).

## Results

### Descriptive analysis. baseline characteristics

A total of 83 PLHIV were included in the analysis, and cardiovascular risk scores were calculated in all participants. Among them, 47 individuals were classified as having high or very high cardiovascular risk and were therefore considered eligible for coronary computed tomography.

The mean age of the participants was 59-years, with a predominance of males (92%). Cardiovascular risk factors were common, with 80% of patients having baseline dyslipidemia, 40% hypertension, 40% active smoking, and 28% type 2 diabetes mellitus; none had a family history of cardiovascular disease. The mean body mass index was 26.25 kg/m^2^ (20–39), and the mean waist circumference was 97.1 cm (83–126). Hepatitis C seropositivity was observed in 28% of patients (Table 1).

**Table 1:** Baseline characteristics of patients undergoing coronary computed tomography.

Variable	
Demographic characteristics	
Age (years)	59
Male %	92.0
Cardiovascular risk factors	
Hypertension %	40.0
Type 2 diabetes mellitus %	28.0
Dyslipidemia at baseline %	80.0
Current smoking %	40.0
Lipid profile at baseline	
Total cholesterol mg/dL	177 (161‒216)
LDL cholesterol mg/dL	121 (99‒133)
HDL cholesterol mg/dL	50 (41‒60)
HIV-related characteristics	
CD4 cell count at baseline, cells/µL	463 (355‒674)
Antiretroviral therapy regimen at baseline	
INSTI-based %	64.0
PI-based %	20.0
Cardiovascular risk scores	
Framingham risk score	21.1 (16.2‒28.6)
SCORE2	7.0 (4.4‒9.0)
REGICOR	5.0 (3.0‒6.0)
D:A:D reduced	10.2 (6.6‒15.6)
D:A:D extended	8.1 (5.8‒14.7)

Continuous variables are expressed as median (interquartile range), and categorical variables as number (percentage). Cardiovascular risk scores are expressed as estimated percentage risk. INSTI, Integrase Strand Transfer Inhibitor; PI, Protease Inhibitor.

The median CD4 cell count was 463 cells/µL, with a mean CD4/CD8 ratio of 0.89. Most patients (64%) were receiving an integrase inhibitor-based antiretroviral regimen, the most frequent being bictegravir/emtricitabine/tenofovir alafenamide (44%), dolutegravir/lamivudine (16%), and rilpivirine/abacavir/lamivudine (8%). All participants had suppressed HIV viral loads.

Metabolic parameters showed a mean fasting glucose of 116.5 mg/dL (82–249), glycated Hemoglobin (HbA1c) of 6.53% (6.0–7.4), and triglyceride-to-glucose index of 8.7 ± 0.63 (range 7.3–10.6). Baseline lipid profiles revealed a mean total cholesterol of 177 mg/dL (103–273), LDL cholesterol of 118.9 mg/dL (35.5–130), HDL cholesterol of 50 mg/dL (26–80), triglycerides of 146.5 mg/dL (65–445), apolipoprotein-B of 86.8 mg/dL (62.5–152), and lipoprotein(a) of 48.1 nmoL/L (7–350), 23.8 mg/dL (4.97–162.30). Lipid-lowering therapy was prescribed in 64% of patients, primarily atorvastatin.

Renal function was preserved, with a mean serum creatinine of 0.96 mg/dL (0.56–1.90) and eGFR of 87.6 mL/min/1.73 m^2^ (55–131.12). Cardiovascular risk scores indicated mean values of 21.1 (16.2‒28.6) for Framingham, 5.0 (3.0‒6.0) for REGICOR, 7.0 (4.4‒9.0) for SCORE2, and 8.1 (5.8‒14.7) for D:A:D.

CAC scoring showed a mean of 184.2 Agatston units (0–1201). CAC showed that 48.0% of the patients had a CAC score of 0, 32.0% had a CAC score between 1 and 100, and 20.0% had a CAC score greater than 100. According to the study's predefined criteria, the 20% of patients were classified as having subclinical coronary artery disease of moderate or high risk.

Among the patients who underwent contrast-enhanced CCTA, evidence of coronary atherosclerosis was identified in 52.2%. Most lesions were mild and non-obstructive, with 34.8% classified as CAD-RADS 1 or 2. More advanced disease was less frequent, with 17.4% presenting CAD-RADS ≥ 3, including only 8.7% with potentially obstructive coronary artery disease (CAD-RADS ≥ 4). 48% of patients had mixed or non-calcified plaques, and 48% exhibited at least one feature of vulnerable or high-risk plaque (34.8% had low-attenuation/lipid-rich plaques, 39.1% showed positive eccentric remodeling, and 43% had spotty calcifications). The “napkin-ring” sign was not observed in any patient.

During follow-up, one patient experienced a lacunar stroke, and two underwent percutaneous coronary revascularization of the left anterior descending artery due to the CT findings.

### Variables associated with the presence of subclinical coronary artery disease

The presence of subclinical coronary artery disease showed a higher and statistically significant association with eccentric plaque remodeling assessed by coronary computed tomography (Mann-Whitney *U*-test, *r* = 0.56, p = 0.003).

Regarding clinical and laboratory variables, moderate associations were identified with fasting glucose levels (Spearman’s rank correlation ρ = 0.47, p = 0.02) and with cardiovascular risk estimated by the Framingham risk score (Spearman’s rank correlation ρ = 0.63, p < 0.001). Moreover, diabetic patients exhibited substantially higher mean coronary calcium scores compared to non-diabetic patients (334 AU, range 0–1201 AU vs. 60.7 AU, range 0–570 AU; t(48) = 4.11, p < 0.001). No statistically significant associations were found between the presence of subclinical coronary artery disease and other clinical, demographic, or laboratory variables evaluated (p > 0.05).

None of the analyzed variables was significantly associated with the development of clinical events.

### Predictive ability of the risk scores

Significant differences in feasibility were observed among the four scales. Differences in the proportion of patients in whom each scale could be calculated were assessed using Cochran’s *Q*-test. Applicability differed significantly among the risk algorithms (*Q* = 364.2; df = 3; p < 0.0001), with the Framingham, REGICOR, and SCORE2 scales being applicable in 98.8%, 97.6%, and 95.3% of patients, respectively, whereas the D:A:D scale could only be calculated in 48% of cases.

Estimated cardiovascular risk varied substantially according to the risk algorithm used. Risk estimates were consistently higher when calculated using the Framingham Risk Score compared with SCORE2, REGICOR, and the D:A:D risk equations, while REGICOR yielded the lowest median risk values. Estimates derived from the D:A:D equation showed intermediate values, with a wider dispersion and lower availability across the cohort (Table 2).

**Table 2:** Distribution of cardiovascular risk estimates by risk score.

Score	Median	Q1	Q3
Framingham	21.10	16.20	28.60
REGICOR	5.00	3.75	6.25
SCORE2	6.70	4.70	08.05
D:A:D reduced	9.32	6.63	15.10
D:A:D	08.07	5.76	14.67

Q1, 25th percentile, lower quartile; Q3, 75th percentile, upper quartile.

Cardiovascular risk estimates were higher in patients with subclinical Coronary Artery Disease (CAC ≥ 100) compared with those with CAC ≤ 100. This trend was observed across all evaluated scores. The difference reached statistical significance only for the Framingham Risk Score (median CAC ≤ 100: 18.3 vs. CAC ≥ 100: 33.4; Welch’s *t*-test, p = 0.049), while SCORE2 (median 6.25 vs. 8.0), REGICOR (4.0 vs. 6.0), D:A:D reduced (8.16 vs. 17.48), and the original D:A:D (7.3 vs. 15.56) showed a similar tendency without statistical significance (Table 3).

**Table 3:** Comparison of cardiovascular risk scores between patients with CAC ≤100 and CAC > 100.

Score	Test	Statistic	p-value	Median CAC ≤ 100	Median CAC > 100
Framingham	Welch’s *t*-test	2.265	0.04	18.3	33.4
REGICOR	Welch’s *t*-test	826	0.42	4.0	6.0
SCORE2	Welch’s *t*-test	1.598	0.16	6.25	8.0
D:A:D reduced	Welch’s *t*-test	1.784	0.15	8.16	17.48
D:A:D	Welch’s *t*-test	1.100	0.38	7.3	15.56

CAC, Coronary Artery Calcium.

**Fig. 2:**
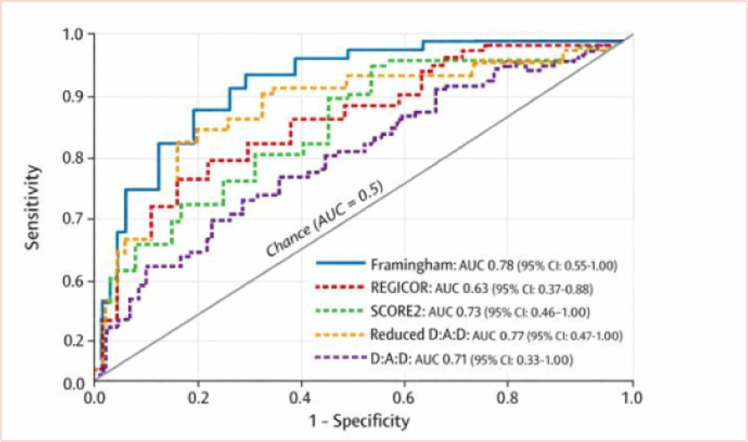
ROC curve comparison of various cardiovascular prediction models. AUC, Area Under the Curve.

A Receiver Operating Characteristic (ROC) curve analysis was performed to evaluate the predictive ability of different cardiovascular risk scores in identifying patients with a Coronary Artery Calcium (CAC) score > 100 (moderate-to-high risk). The Framingham Risk Score demonstrated the highest discriminative ability (AUC = 0.78; 95% CI 0.55–1.00), followed by the reduced D:A:D score (AUC = 0.77; 95% CI 0.47–1.00). SCORE2 (AUC = 0.73; 95% CI 0.46–1.00), the original D:A:D (AUC = 0.71; 95% CI 0.33–1.00), and REGICOR (AUC = 0.63; 95% CI 0.37–0.88) showed lower discriminative ability in this sample. ROC curves for each risk score are shown in Fig. 2, and detailed values are presented in Table 4.

**Table 4:** Discriminative performance of cardiovascular risk scores for predicting CAC > 100.

SCORE2	AUC	95% IC	p
Framingham	0.78	0.55 – 1.00	0.06
REGICOR	0.63	0.37 – 0.88	0.40
SCORE2	0.73	0.46 – 1.00	0.15
Reduced D:A:D	0.77	0.47 – 1.00	0.17
D:A:D	0.71	0.33 – 1.00	0.30

CI, Confidence interval; AUC, Area Under the Curve.

## Discussion

Numerous studies have identified both traditional cardiovascular risk factors and HIV-specific factors associated with the development of coronary artery disease in PLHIV. HIV-specific determinants include the chronic inflammatory state induced by the infection, high viral load, low CD4 cell counts, co-infections such as hepatitis C, and prolonged exposure to ART, particularly protease inhibitors. In the studied cohort, HIV-related factors were not associated with coronary artery disease, likely due to adequate virological control, as all patients had undetectable viral loads and a median duration of ART of 10.5-years (7.25–28). Overall, the cohort consisted of young patients with relatively short-standing infection and good disease control. Regarding traditional cardiovascular risk factors, diabetes was significantly associated with coronary artery disease in the present cohort. Twenty-eight percent of the patients were diabetic and had adequate glycemic control, with a median fasting glucose of 149 mg/dL and a median HbA1c of 6.5%. Despite this control, coronary calcium scores were significantly higher in diabetic patients compared to non-diabetic patients (mean = 334 AU, range 0–1201 AU vs. mean 60.7 AU, range 0–570 AU, t(48) = 4.11, p < 0.001), highlighting the importance of diabetes as a risk factor even when well controlled. On the other hand, variables such as well-controlled hypertension, relatively young age, absence of obesity, and the low proportion of women likely contributed to the lack of association of these factors with coronary disease. Of particular note is the lipid profile, as none of its components (total cholesterol, LDL, HDL, triglycerides, Apo B, or lipoprotein(a)) were associated with coronary artery disease. This may be because lipid abnormalities require more time to cause endothelial damage in the coronary arteries, or because the studied patients had well-controlled lipid levels, with 64% receiving chronic statin therapy and a mean LDL cholesterol of 118.9 mg/dL (35.5–130) or apolipoprotein B of 86.8 mg/dL (62.5–152).

Cardiovascular risk prediction in PLHIV remains challenging. Traditional risk scores such as Framingham, SCORE2 and REGICOR were developed in HIV-negative populations and may not adequately incorporate HIV-related determinants of cardiovascular disease, including chronic inflammation, immune dysregulation and cumulative antiretroviral exposure. Other studies comparing general population risk equations with HIV-specific tools have shown limited agreement with markers of subclinical atherosclerosis. Serrano-Villar S et al.^34^ demonstrated that the D:A:D risk equation had a slightly better ability to identify subclinical vascular disease than the Framingham or SCORE equations; however, the evaluation was based on carotid intima-media thickness rather than coronary imaging, and overall discrimination remained modest.^34^ Similarly, longitudinal analyses indicate that they still underestimate the burden of subclinical atherosclerosis in this population. These observations are concordant with the present results, reinforcing the notion that conventional cardiovascular risk algorithms do not fully capture the true extent of coronary disease in PLHIV.^24^^,^^35^

In the present study, the authors assessed the relationship between cardiovascular risk estimates derived from commonly used clinical risk scores and subclinical coronary artery disease as evaluated by coronary computed tomography in PLHIV classified as having high or very high cardiovascular risk. Although patients with subclinical coronary disease had higher estimated risk values than those without, the predictive capacity of the scores was moderate. The Framingham score showed a better ability to predict the presence of moderate-to-high risk coronary disease compared with other risk scores. Notably, this finding is significant, as it did not correspond to either SCORE2, commonly used by cardiology specialists, or the D:A:D score, which incorporates the largest number of HIV-related variables. While the authors cannot rule out that the D:A:D score may be more accurate, it was completed in only 48% of patients, which limits its statistical analysis. Although the D:A:D score is HIV-specific, its real-world utility is severely limited by the lack of historical data, whereas traditional scores, although less specific, can almost always be calculated. The low proportion of patients in whom it was completed is due to the difficulty of collecting the variables required for its calculation in routine clinical practice. The Framingham score was the one that could be completed in the highest proportion of patients during the study visit.

CCTA provides information that extends beyond traditional risk scores by directly characterizing coronary atherosclerotic burden and plaque phenotype. In PLHIV, several contemporary imaging studies have demonstrated a higher prevalence of non-calcified coronary plaque and complex plaque features compared with non-HIV populations, even among individuals with low or absent coronary calcium. For instance, data from large cohorts show that asymptomatic PLHIV have a greater prevalence of any coronary plaque and of non-calcified components despite similar or lower CAC scores, suggesting that reliance on calcium scoring alone may underestimate total atherosclerotic burden in this setting.^9^ These imaging characteristics underscore the added value of CCTA for comprehensive assessment in high-risk HIV cohorts and support its potential role as a complementary risk stratification tool, which may be particularly relevant in clinical scenarios where risk estimation is uncertain.

Among the patients who underwent contrast-enhanced coronary computed tomography angiography, evidence of coronary atherosclerosis was identified in 52.2%. Most lesions were mild and non-obstructive, with 34.8% classified as CAD-RADS-1 or −2. More advanced disease was less frequent, with 17.4% presenting CAD-RADS ≥3, including only 8.7% with potentially obstructive coronary artery disease (CAD-RADS ≥4). 48% showed plaques with vulnerability features. Overall, this represents a population with a high prevalence of soft and vulnerable plaques, consistent with previously published literature.

No clinical, laboratory, or imaging variable was associated with the events, most likely due to the low total number of events (n = 3) and the short follow-up of one year.

Several limitations of this study should be acknowledged. First, the sample size was relatively small, particularly with respect to patients with significant coronary atherosclerosis, which may have limited statistical power and reduced the ability to detect subtle differences between risk scores. Second, coronary computed tomography was performed exclusively in individuals classified as having high or very high cardiovascular risk, which may restrict the generalizability of these findings to lower-risk populations. Third, the clinical follow-up of the patients was limited to only one year, which is an insufficient period to adequately assess the occurrence of major cardiovascular events or other relevant clinical outcomes. Finally, not all cardiovascular risk equations could be calculated in all participants due to incomplete clinical or historical treatment data, which may have influenced comparative performance across tools. In view of these constraints, the present results should be interpreted with caution and considered within the broader context of existing clinical research, highlighting the need for larger, prospective studies to further clarify cardiovascular risk assessment in PLHIV.

In conclusion, this study provides contemporary real-world data on the relationship between commonly used cardiovascular risk scores and subclinical coronary artery disease detected by coronary computed tomography in PLHIV considered to be at high or very high cardiovascular risk. These findings suggest that, in current clinical practice, traditional risk tools may not adequately reflect the complexity of cardiovascular risk in this population and support the potential complementary value of coronary imaging in selected high-risk individuals. Future larger studies with longer follow-up are needed to further validate the role of CCTA in guiding therapy in this population.

## Conclusions

In PLHIV with virological suppression and multiple cardiovascular risk factors, subclinical coronary atherosclerosis was highly prevalent (52.2%), even in the absence of prior clinical cardiovascular disease. Cardiac CT proved useful for its detection. A substantial proportion of coronary plaques were vulnerable (48%), characterized by lipid-rich content, spotty calcifications, and positive remodeling. Subclinical coronary artery disease was mainly associated with higher fasting glucose levels, highlighting the continuous relationship between dysglycemia and atherosclerosis in this population. Cardiovascular risk scores showed variable applicability and predictive performance, with the Framingham score best identifying patients with CAC > 100. The D:A:D risk score could only be calculated in less than half of the patients, limiting its usefulness in routine clinical practice.

## Data availability statement

All data generated or analyzed during this study are included in the text.

## Reporting guideline

This manuscript adheres to the STROBE guidelines for observational studies (cohort, case-control, and cross-sectional studies).

## Conflicts of interest

All authors have approved the submitted version and declare no conflicts of interest related to this work. The study was conducted in accordance with institutional ethical standards and the Declaration of Helsinki, and written informed consent was obtained from all participants.
